# Dental injuries in paediatric mandibular fracture patients

**DOI:** 10.1007/s10006-021-00966-8

**Published:** 2021-04-28

**Authors:** Leena Kannari, Emilia Marttila, Hanna Thorén, Johanna Snäll

**Affiliations:** 1 Department of Oral and Maxillofacial Diseases, University of Helsinki and Helsinki University Hospital, 00029 Helsinki, Finland; 2 Department of Oral and Maxillofacial Surgery, Institute of Dentistry, University of Turku, Turku, Finland; 3 Department of Oral and Maxillofacial Diseases, Turku University Hospital, Turku, Finland

**Keywords:** Dental injury, Paediatric patient, Mandibular fracture, Tooth loss

## Abstract

**Purpose:**

Dental injuries (DIs) are associated with facial fractures, particularly mandibular fractures. As paediatric mandibular fractures have special features, we sought to clarify the occurrence and types of DIs among this patient group. We assessed how age, injury type, and fracture location affects the occurrence of DIs and thereby defined which patients are most susceptible.

**Methods:**

This retrospective study included patients < 18 years with a recent mandibular fracture. Predictor variables were gender, age group, mechanism of injury, type of mandibular fracture, and other associated facial fracture(s). Types and locations of DIs and tooth loss due to injury were also reported.

**Results:**

DIs were detected in 34.7% (*n* = 41) out of 118 patients. Patients with tooth injury had on average 3.5 injured teeth. A total of 16.2% of injured teeth were lost, typically at the time of the injury. Loss of at least one tooth was seen in approximately 10% of patients. Avulsion was the most common cause of tooth loss (52.2%). Non-complicated crown fracture (50.7%) was the most common DI type. Statistically significant associations between studied variables and DIs were not detected.

**Conclusion:**

DIs are common and often multiple in paediatric mandibular fracture patients regardless of background factors. DIs often lead to tooth loss. Prompt replantation of an avulsed tooth, early detection of DIs, and prevention of tooth loss whenever possible are important to avoid permanent tooth defects.

## Introduction

The occurrence of traumatic dental injuries (DIs) in patients with facial fractures varies between 13.1 and 22.5% [1–3]. The presence of DIs is especially high in paediatric patients with facial fractures [1, 3–5] and occurs in 23% [1] to 31% [4] of patients. DIs are particularly associated with mandibular fractures (39%) [5], with a corresponding rate of 29% in the paediatric population [1]. Most (62%) DIs occur in permanent teeth [6]. These irreversible injuries lead to permanent disadvantages throughout adolescence and adulthood.

More than half of reported DIs in patients with facial fractures are crown or root fractures [4, 5]. In addition, avulsions and luxations are fairly frequent [2, 5]. The majority of DIs are easy to detect at the primary assessment by clinical examination and supplementary radiological imaging. However, root fractures and delayed pulp necrosis in particular require closer examination and long-term follow-up [7, 8]. The paediatric patient’s lack of cooperation and the characteristics of developing dentition may also complicate the detection of DIs and thereby hinder initiation of treatment.

DIs may lead to numerous dental visits and long-term rehabilitation of occlusion. Final reconstructions with dental implants, if needed, are not recommended before the end of adolescence [9]. Thus, DIs may affect self-esteem and psychological well-being, especially in teenagers. This highlights the importance of the best possible treatment of DIs.

The purpose of this study was to evaluate the occurrence and types of DIs in mandibular fracture patients among children and adolescents. We sought to clarify how age, injury type, and fracture location affect the occurrence of DIs and how this information may identify which patients are most susceptible to DIs and would benefit from further assessments by dentists specialized in tooth injuries. We hypothesized that DIs are common in the present facial fracture population and routine collaboration with dentists treating paediatric injuries may be necessary.

## Patients and methods

### Study design

The records of all patients < 18 years presenting at the Emergency Unit of Oral and Maxillofacial Surgery at Helsinki University Hospital with a recent mandibular fracture between 1 January 2013 and 31 December 2018 were retrieved from electronic patient records. All injury-related patient records were assessed retrospectively.

The following data were recorded from the patient files: age, sex, injury mechanism, type of mandibular fracture(s), possible associated facial fracture(s), injury-induced DIs, and duration of follow-up.

### Study variables

The outcome variable was DI and was defined as any clinically or radiologically (or both) detected injury of the dentition that had been caused by the trauma leading to the mandibular fracture. DIs were detected at either the first health care contact or during the further injury follow-ups.

The predictor variables were sex, age group, mechanism of injury, type of mandibular fracture, and other associated facial fracture(s). Age was stratified into one of the following subgroups: (1) < 7 years, (2) between 7 and 12 years, (3) between 13 and 15 years, and (4) between 16 and 17 years. Injury mechanisms were grouped into the following seven categories: (1) assault, (2) ground-level fall, (3) bicycle accident, (4) traffic accident, (5) sports accident, (6) fall from height, and (7) other (i.e. none of the previous six). Fracture(s) of the mandible were further classified as (1) tooth-bearing region fracture(s); (2) non-tooth-bearing region fractures (i.e. fracture[s] of the mandibular condyle, ramus, or both); or (3) combined fractures (i.e. combination of the previous two).

Types and locations of the DIs and tooth loss were reported. The main type of each DI was categorized as non-complicated crown fracture, complicated fracture, avulsion, luxation, intrusion, swinging tooth (i.e. a tooth with increased mobility caused by injury), and other (i.e. none of the previous six, for example contusion leading to toothache, pulp necrosis, or both during the follow-up). In addition, the duration of the follow-up period in the hospital, hospital outpatient care, or both was reported.

### Statistical analyses

Data were analysed using GraphPad Prism version 5.00 (GraphPad Inc.). The two-tailed Mann–Whitney test was used to assess the significance of the differences in continuous variables. Fisher’s exact test was used to examine the association between variables with nominal scales. *p*-values < 0.05 were considered statistically significant.

## Results

Data from a total of 118 patients < 18 years with mandibular fractures were included and analysed. Descriptive statistics of the patients are presented in Table 1. The mean age of the patients at the time of injury was 13.3 years (range 0.5–17.9, median 14.6 years). Most patients were male (72.9%). The most common fracture mechanism was ground-level fall (23.7%) followed by bicycle accident (22.9%). Over half of the patients had fractures involving a non-tooth-bearing region (56.8%). Follow-up durations in the hospital or hospital outpatient clinic ranged from 1 week to 3 years. Most patients (83.1%) were followed for a minimum of 1 month.

**Table 1:** Descriptive statistics of 118 patients with mandibular fracture

Age	Years	
Range	0.5–17.9	
Mean	13.3	
Median	14.6	
Gender	Number of patients	% of 118
Male	86	72.9
Female	32	27.1
Age (years)		
< 7	15	12.7
≥ 7 to < 13	30	25.4
≥ 13 to < 16	28	23.7
≥ 16 to < 18	45	38.1
Mechanism of injury		
Ground-level fall	28	23.7
Bicycle accident	27	22.9
Assault	18	15.3
Traffic accident	14	11.9
Sports accident	14	11.9
Fall from height	12	10.2
Other	5	4.2
Mandibular fracture type		
Tooth-bearing region	16	13.6
Non-tooth-bearing region	67	56.8
Combined	35	29.7
Mandible with other facial fracture		
Yes	13	11.0
No	105	89.0

DIs were detected in 41 of the 118 patients (34.7%). The total number of injured teeth was 142. Differences between studied variables in patients with and without DIs remained statistically non-significant (Table 2). However, 23 of 41 patients with DIs (56.1%) had fractures exclusively in the non-tooth-bearing regions, whereas only 4 out of 41 (9.8%) had fractures exclusively in the tooth-bearing regions.

**Table 2:** Statistics of 118 patients with and without dental injury

	*n*	Dental injury: yes	% of *n*	Dental injury: no	% of *n*	
All	118	41	34.7	77	65.3	
Sex						
Male	86	29	33.7	57	66.3	*p* = 0.8282
Female	32	12	37.5	20	62.5	
Age group						
< 7	15	6	40.0	9	60.0	*p* = 0.7433
≥ 7 to < 13	30	12	40.0	18	60.0	
≥ 13 to < 16	28	10	35.7	18	64.3	
≥ 16 to < 18	45	13	28.9	32	71.1	
Mechanism of injury						
Ground-level fall	28	13	46.4	15	53.6	*p* = 0.1862
Bicycle accident	27	11	40.7	16	59.3	
Assault	18	2	11.1	16	88.9	
Traffic accident	14	6	42.9	8	57.1	
Sports accident	14	3	21.4	11	78.6	
Fall from height	12	5	41.7	7	58.3	
Other	5	1	20.0	4	80.0	
Mandibular fracture type						
Tooth-bearing region	16	4	25.0	12	75.0	*p* = 0.5765
Non-tooth-bearing region	67	23	34.3	44	65.7	
Combined	35	14	40.0	21	60.0	
Mandible with other facial fracture						
Yes	13	5	38.5	8	61.5	*p* = 0.7652
No	105	36	34.3	69	65.7	

Overall, there were 112 injured permanent teeth and 30 injured deciduous teeth. The number of injured teeth ranged from 1 to 11 (mean 3.5). DIs were equally frequent in the lower and upper jaw, with 71 DIs in each. Non-complicated crown fracture was the most common DI type in both permanent (51.7% of 112 permanent teeth) and deciduous teeth (42.6% of 30 deciduous teeth), followed by complicated fracture in permanent teeth (15.2% of 112 permanent teeth) and avulsion in deciduous teeth (33.1% of 30 deciduous teeth). Nine patients had combinations of several types of injuries, such as crown fracture and luxation. Intruded teeth were rare and occurred in only 1.4% of the patients. Nine of the total 20 avulsed teeth were replanted during primary care.

**Fig. 1:**
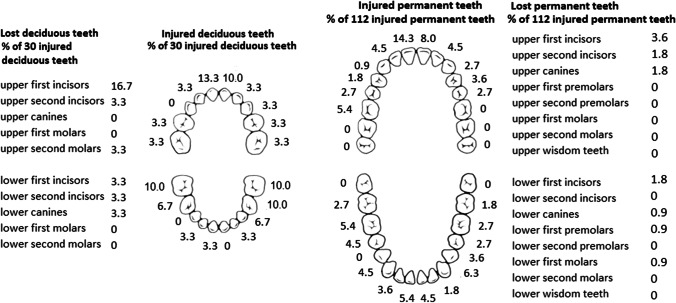
Locations of injured and lost teeth in children and adolescents with mandibular fractures

Of all 142 tooth injuries, the majority were observed in permanent upper incisors (17.6%), permanent lower canines (8.5%), and permanent lower incisors (7.7%). Of 30 injured deciduous teeth, upper incisors were injured most often (23.3%) (Fig. 1).

Tooth loss occurred in 12 patients (10.2%) and in 23 of 142 injured teeth (16.2%). Of lost teeth, 13 were permanent and 10 were deciduous teeth. Most lost teeth (18 out of 23, 78.3%) were lost at time of injury or immediately after primary care. The remaining 5 teeth were lost during further follow-up (range 3 months to 3.5 years, mean 2.6 years, median 1.75 years).

**Fig. 2:**
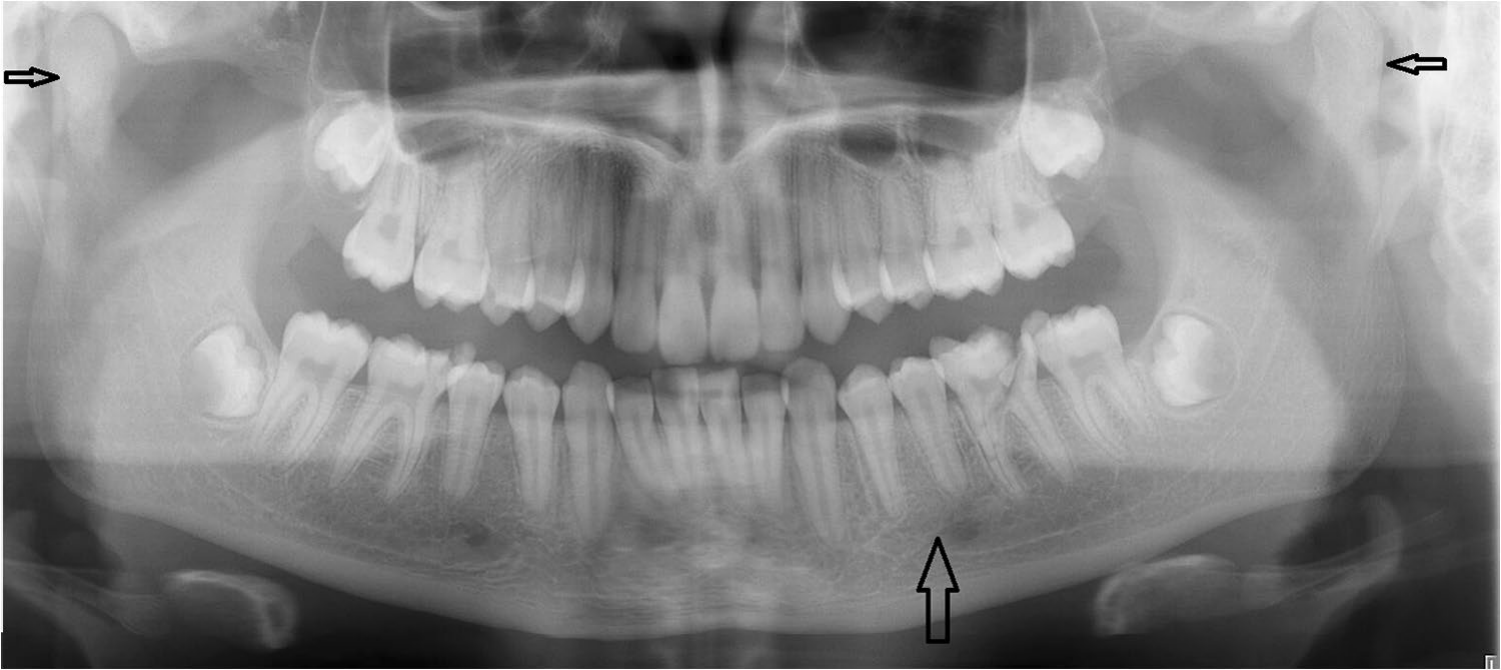
A 14-year-old girl suffered bilateral condylar fractures with an additional symphysis fracture of the mandible and multiple dental injuries due to fainting and ground-level fall. A dental panoramic radiograph image shows a sagittal corpus fracture (wide arrow) and bilateral condyle fractures (small arrows) of the mandible, which were more detectable with additional imaging. Fractures were treated by intermaxillary fixation and a soft diet. A fragmentary crown-root fracture was observed in the lower left first molar that was removed under general anaesthesia at primary fracture treatment. Additionally, crown fractures were observed in the upper right second premolar and lower right first molar. The lower left premolars did not respond to vitalometer after injury, which was partially explained by the fracture-related neurosensory disturbance in mandibular inferior nerve. Further dental follow-up revealed periapical signs of devitalization and the patient received root treatment to the lower second premolar 14 months after injury. Tooth loss was replaced with an implant at the end of the patient’s growth

Upper incisors were lost most often (Fig. 1). Reasons for tooth loss included avulsion or failed replantation of an avulsed tooth (*n* = 12, 52.2% out of 23 teeth lost), complicated root or crown fractures, or both (*n* = 5, 21.7% out of 23 lost teeth) (Fig. 2), and one intrusion (4.3% out of 23 lost teeth). The remaining 5 teeth were lost due to periodontal ligament injury, with or without pulp necrosis, and were situated in the fracture line (21.7% out of 23 teeth).

## Discussion

The purpose of this study was to evaluate the occurrence and types of DIs in paediatric patients with mandibular fracture. We hypothesized that DIs are common in these patients and routine collaboration with dentists treating paediatric injuries may be necessary. Our hypothesis was confirmed. DIs were common regardless of background factors and occurred in more than a third (34.7%) of the patients. Up to 16% of damaged teeth were lost and most of these (78.3%) were lost at the primary injury stage. DIs were often multiple and one or more teeth were lost in every tenth patient.

Traumatic tooth loss was usually seen in the upper incisive region. The number of avulsed teeth was also rather high; 52.2% of lost teeth were due to avulsion, failed replantation of an avulsed tooth, or both. However, it should be noted that only 22% of the replanted teeth were lost during the follow-up. Thus, early immediate replantation of a permanent tooth may result in successful or at least long-term benefit. Sometimes, the consequences of dental accidents must be treated after years or even decades. Although replacement of lost teeth for appearance would be important, dental implant reconstruction is not recommended until the end of growth. Premature implantation can lead to complications, such as infraocclusion and rotation of dental implants [9]. Therefore, the importance of early replanting should be emphasized in paediatric patients. As it is known that teeth have a considerable effect on appearance [10, 11], these injuries may affect patients in different ways throughout their childhood and teenage years.

Patients with combined mandibular and other facial fractures suffered from DIs more frequently than patients with an isolated mandibular fracture. Injuries causing multiple facial fractures are often due to high-energy trauma. However, although not statistically significant, a notable finding was that over half (56.1%) of the patients who had DIs had fractures only in the ascending part of the mandible, indicating that DIs frequently occur indirectly as a result of forceful closure of the lower jaw. Surprisingly, DIs were infrequently associated with fractures in tooth-bearing areas. On the other hand, teeth in the fracture line are prone to pulp or ligament injuries (or both), which may occur with a delay [12] and be asymptomatic [13]. Therefore, teeth in the fracture line in particular should be followed from months to years [14].

The rate of DIs in this study is consistent with previous studies [1, 3, 5, 15]. Although Lieger et al. observed the highest rates in adolescents [5], in the present study with a larger number of patients, DIs were more prevalent in younger age groups, although no statistical significance was observed. The changing proportions of the facial regions during growth may explain the prevalence of DIs in younger age groups. The lower jaw and teeth form a significant part of the face in children, whereas the midfacial region and sinuses grow to a greater proportion of the face with increasing age. Additionally, motor skills are still developing and facial protection at time of injury may be deficient even though playing, climbing, and all forms of movement are integral parts of daily life. Primary teeth are also a slightly softer than permanent teeth [16, 17] and are thus more prone to fractures. Overall, in the mandibular fracture population, the youngest children were more prone to DIs than older age groups for several reasons.

Even if non-complicated crown fracture was the most common DI type in this study, consistent with previous studies [1, 5], DIs were often multiple. Further DI treatment may require numerous visits and general anaesthesia may be required for children. Additionally, neurosensory disturbances may create challenges in endodontic diagnosis. Regular comprehensive clinical examinations combined with radiological evaluations are often required, for up to 5 years after injury depending on the tooth injury [7, 13, 14].

Limitations of this study include the retrospective nature and range of the follow-up period. Long-term outcomes are thus underrepresented in studies focusing on fractures. Therefore, DI occurrence in the present study is probably underestimated due to the varying follow-up periods in our unit.

## Conclusions

The present study highlights the frequency and severity of DIs in paediatric and adolescent patients with mandibular fractures. DI patients had an average of 3.5 injured teeth. In 29.3% of DI patients, the injury resulted in tooth loss, which typically occurred at the time of injury or during the immediate treatment of the fracture. Thus, DIs are not only common but are also often severe and lead to tooth loss. Prompt replantation of avulsed teeth as soon as possible, preferably in less than an hour [18], as well as early careful dental evaluation, and systematic practices for further follow-up can be recommended for all paediatric patients with mandibular fractures.

## Data Availability

Data are available on request.
